# AGING SCIENCE EDUCATION: OLLI COLLABORATION BENEFITS UNDERREPRESENTED STUDENTS

**DOI:** 10.1093/geroni/igae098.3099

**Published:** 2024-12-31

**Authors:** Maeva Laflamme, Melanie Horn Mallers, Laura Zettel-Watson, Jennifer Piazza

**Affiliations:** California State University Fullerton, Fullerton, California, United States; California State University Fullerton, Fullerton, California, United States; California State University Fullerton, Fullerton, California, United States; California State University Fullerton, Fullerton, California, United States

## Abstract

The aging population is growing and diversifying, generating a need for workforce specialists (e.g., scientists, physicians) prepared to integrate knowledge about aging into their respective fields. Unfortunately, the fields that bring innovative change to improve the lives of older adults are the fields that continue to lack diversity due to a “leaky pipeline,” whereby underrepresented students are often lost or pushed out academically from MSTEM fields. To increase diversity in the aging-science workforce, a critical goal is to implement training programs that advance the study of aging within MSTEM. With the support of NIH funding, we designed an empirically derived, multi-pronged program to purposefully intersect the study of aging with science and research. To increase student involvement with older adults, we partnered with our campus-based Osher Lifelong Learning Institute (OLLI) members, many of whom are scientists themselves, regularly serve as panelists, interview participants, proofread students’ works, and act as audience members for our students. They also informally interact with students through community-based service learning opportunities, outreach, and volunteer activities. After collaborating with OLLI for one year, students report greater empathy and joy when interacting with older adults, increased interest and motivation to work in aging sciences, and decreased ageism. The MSTEM-OLLI collaboration has served as a critical component of advancing our goal of recruiting, retaining, and preparing underrepresented undergraduates for a future in MSTEM aging research. Our findings indicate that strengthening and expanding meaningful partnerships between older adults and students is a key priority to enhancing aging science programs.

